# The mutated in colorectal cancer (*MCC*) gene can serve as a potential biomarker of glioblastoma

**DOI:** 10.3389/fonc.2024.1435605

**Published:** 2024-10-08

**Authors:** Huonggiang Nguyen, Qingzhi Huang, Uijin Juang, Suhwan Gwon, Woohyeong Jung, Soohyeon Lee, Beomwoo Lee, So Hee Kwon, In Soo Kim, Jongsun Park, Seon-Hwan Kim

**Affiliations:** ^1^ Department of Pharmacology, College of Medicine, Chungnam National University, Daejeon, Republic of Korea; ^2^ Department of Medical Science, Metabolic Syndrome and Cell Signaling Laboratory, Institute for Cancer Research, College of Medicine, Chungnam National University, Daejeon, Republic of Korea; ^3^ College of Pharmacy, Yonsei Institute of Pharmaceutical Sciences, Yonsei University, Incheon, Republic of Korea; ^4^ Department of Neurosurgery, Institute for Cancer Research, College of Medicine, Chungnam National University, Daejeon, Republic of Korea

**Keywords:** MCC, glioblastoma, cancer, biomarker, brain

## Abstract

**Introduction:**

The mutated in colorectal cancer (MCC) gene was initially identified as a candidate tumor suppressor gene in colorectal cancer, acting as a negative regulator of cell cycle progression. However, its functional roles in brain tumors, particularly glioblastoma, remain largely unexplored. This study reveals a significant association between MCC status and glioblastoma.

**Methods:**

We explored MCC expression in the glioblastoma database, patient samples, and cell lines. We investigated the proliferation and migration of the cell lines in MCC gene knockdown using small interfering RNA.

**Results:**

*In vitro* analyses revealed elevated protein and mRNA levels of MCC in several glioblastoma cell lines (U118MG and T98G). Silencing MCC expression via siRNA-mediated knockdown resulted in increased proliferation and migration of these cell lines. Supporting these findings, analyses of The Cancer Genome Atlas (TCGA), Chinese Glioma Genome Atlas (CGGA), and Genotype-Tissue Expression (GTEx) databases confirmed higher MCC expression in glioblastoma tumors than in normal brain tissue. Importantly, we observed that high MCC expression was associated with poor prognosis in glioblastoma patients, highlighting its potential role in disease progression. Additionally, this study identifies a nuclear localization of MCC in the glioblastoma cell line.

**Discussion:**

These findings indicate that MCC expression is significantly upregulated in glioblastoma and may play a role in its pathophysiology, warranting further investigation.

## Introduction

Glioblastoma IDH-wildtype, the most prevalent primary intracranial malignancy, is associated with a very low 5-year survival rate (6.8% in 2023) (1). Glioblastoma arises predominantly in the cerebral hemispheres, with approximately 95% of all cases occurring in the supratentorial region; significantly fewer tumors occur in the cerebellum, brainstem, and spinal cord (2). To accurately determine tumor location, it is essential to employ advanced imaging technologies, including computed tomography (CT, also known as “CAT scan”) and magnetic resonance imaging (MRI) (3). According to cIMPACT-NOW [the consortium that makes recommendations on molecular and practical approaches to central nervous system (CNS) tumor classifications] (4) and the fifth edition of the World Health Organization (WHO) Classification of Tumors of the Central Nervous System (WHO CNS5) (2021) (5), glioblastoma is no longer divided into isocitrate dehydrogenase (IDH)-wild-type and IDH-mutant groups; all glioblastomas are now classified as wild-type IDH. Glioblastoma is currently considered a diffuse astrocytic glioma classified as IDH-wild-type or H3-wild-type. Glioblastoma exhibits one or more of the following histological and genetic hallmarks: microvascular proliferation, necrosis, mutation of the telomerase reverse transcriptase (TERT) promoter, amplification of the epidermal growth factor receptor (*EGFR*) gene, and chromosomal alterations such as +7/−10 copy number changes. Based on these features, glioblastoma is categorized as a grade 4 glioma according to the 2021 WHO CNS5 criteria (5). Given the revised definition of glioblastoma, it has become evident that biomarkers play pivotal roles in characterizing the disease. In addition, to improve diagnostic accuracy, sequencing that aids molecular analysis can also be conducted (6). The current standard of care for glioblastoma involves a multimodal approach that starts with surgical resection for optimal tumor removal. This is followed by adjuvant therapy, which typically includes a combination of radiation therapy and concurrent administration of temozolomide. This regimen is designed to target residual tumor cells, with the goal of improving survival outcomes and attenuating disease progression (7). Even though many treatments involve surgery, radiation, and chemotherapy, biomarkers that aid accurate and early diagnosis remain limited (8). Diagnostic biomarkers ensure more precise tumor classification (9).

The mutated in colorectal cancer (*MCC*) gene, located on the long arm of chromosome 5 (5q21), encodes a multifunctional protein also known as MCC (10). The discovery of MCC in 1991 was pivotal, and it served as a tumor suppressor in familial adenomatous polyposis (FAP) (10, 11). Further research has revealed the role played by MCC in inhibiting cell cycle progression, not only in NIH3T3 fibroblasts but also in colorectal cancer (CRC), highlighting the significance of MCC in regulating cell growth and its potential effects on cancer development (12, 13). Additionally, in CRC samples, MCC blocked cell migration and proliferation (13). In Purkinje cells and nerve fibers, MCC has been located in both the plasma membranes and membrane organelles (12). In this study, the potential role of MCC in the pathophysiology of glioblastoma was investigated. The expression of MCC in human glioblastoma cell lines, normal brain tissue, and tumor tissues was analyzed, and experiments were performed to elucidate the function of MCC in glioblastoma cells. This information increases our understanding of the biological mechanisms underlying brain cancer progression.

## Materials and methods

### Human samples

The study was approved by the Hospital Institutional Review Board (approval number CNUH 2013-11-006) following the Declaration of Helsinki at Chungnam National University Hospital (Daejeon, South Korea), and written informed consent was obtained from all patients before surgery. Normal brain tissue samples were obtained from cadavers by autopsies of the surrounding normal brain of glioblastoma patients who underwent surgery.

### Cell culture and transfection

The glioblastoma cells (U118MG and T98G) were maintained in Dulbecco’s modified Eagle medium supplemented with 10% fetal bovine serum and 1% Antibiotics-Antimycotics (Life Technologies, Carlsbad, CA, USA). U118MG cells and T98G cells were transiently transfected with 30 nM scrambled siRNA (sc-37007) (Dharmacon, Lafayette, CO, USA) or MCC siRNA (sc-106908) (Dharmacon, Lafaynette, CO, USA) using Lipofectamine 3000 (Invitrogen, Carlsbad, CA, USA).

### Immunoblot analysis

As described previously (14), cells were placed on ice for harvest and lysis in lysis buffer (PRO-PREP™ Protein Extraction Solution, cat. No 17081, iNtRON Biotechnology, South Korea), and lysates were centrifuged for 20 min at 12,000 rpm. The cell lysates were resolved by 7.5–12.5% SDS-PAGE and then transferred to Immobilon-P membranes (Millipore). The membranes were blocked for 1 h in 1 × tri-buffered saline buffer, including 5% skimmed milk and 0.1% Tween 20 (TBST), followed by overnight incubation with the anti-MCC, anti-β-actin, and anti-GAPDH antibodies diluted in 3% bovine serum albumin at 4°C. The secondary antibody was horseradish peroxidase-conjugated anti-rabbit IgG or anti-mouse IgG (Invitrogen, Carlsbad, CA, USA), diluted 5,000-fold in the blocking buffer. Visualization was achieved with chemiluminescence through X-ray film exposure by enhanced chemiluminescence (ECL) (ProNA™ECL Ottimo, TransLab, Daejeon, South Korea) detection.

### Real-time quantitative reverse transcription-polymerase chain reaction

Total RNA in cells was isolated using TRIzol (Invitrogen, Grand Island, USA) to synthesis cDNA using the SuperScript III First-Strand Synthesis System for qPCR (Invitrogen, Grand Island, USA). To measure duplex DNA formation, qPCR measurements of individual cDNAs were performed using SYBR green dye. The reactions were conducted in triplicate with the GoTaq^®^ qPCR Master Mix from Promega (St. Woods Hollow Madison, USA) and normalized to the expression of β-Actin mRNA. The following primers were used in the qPCR: MCC forward, 5′-TACGAATCCAATGCCACA-3′; MCC reverse, 5′-AGCTTCATGAGCAGGGCCTT-3′; Actin forward, 5′-TCACCCACACTGTGCCCATCTACGA-3′; and Actin reverse, 5′-CAGCGGAACCGCTCATTGCCAATGG-3′.

### Immunofluorescence analysis

U118MG and T98G cells were grown on the glass coverslips until they reached 60–70% confluence. After 24 h, the cells were fixed in 4% paraformaldehyde for 14 min and then permeabilized in 0.2% Triton X-100 for 5 min at room temperature. Then, cells were incubated in a blocking buffer containing 5% bovine serum albumin (Sigma, Livonia, Michigan, USA) in TBST for 1 h at 37°C. The first antibody MCC (Santa Cruz Biotechnologies, Dallas, Texas, USA) was diluted 100-fold for the primary antibody and incubated overnight. Alexa Fluor 647-conjugated anti-mouse IgG antibody (Invitrogen, Carlsbad, CA, USA) was used. After several rinsing times, coverslips were mounted using Vectashield (Vector Laboratories Newark, CA, USA) and visualized using a ZEISS LSM 900 confocal microscope.

### Immunohistochemistry

Paraffin sections were de-paraffinized several times with xylene and then rehydrated in a graded series of ethanol solutions. Then, slides were deep in peroxidase for 20 minutes to reduce nonspecific background staining. After the slides were washed twice with PBS for 10 min, 10% goat serum (cat No. S-1000-20, Vector Laboratories, Newark, CA, USA) was added for blocking, followed by incubation for 1 h. Following three washes with PBS, the primary antibody was applied at 4°C overnight. Then, biotinylated secondary antibody was diluted in blocking buffer after rinsing with PBS, and the slides were incubated at room temperature for 1 h. The slides were rinsed with PBS and placed in VECTASTAIN^®^ Elite^®^ ABC-HRP Reagent, Peroxidase, R.T.U. (cat No.PK-7100, Vector Laboratories, Newark, CA, USA). After the slides were washed with PBS, DAB chromogen and a mixture of reagents 1, 2, and 3 (Vector Laboratories, Newark, CA, USA) were added, and signals were observed with a microscope. The reaction was stopped with distilled water, coverslips were mounted with Permount mounting medium (Fisher Chemical, PA, USA), and images were taken with an EVOS M5000 Imaging System. The positive staining area was analyzed using ImageJ software (ImageJ, United States).

### Bioinformatics data set

Data related to MCC expression levels in cell lines were collected from The Human Protein Atlas. The pan-cancer analysis data were downloaded from Gene Expression Profiling Interactive Analysis, where glioma datasets were obtained from publicly available databases such as the Cancer Genome Atlas Project (TCGA) and Genotypic-Tissue Expression (GTEx). Gene methylation analysis was performed using the Chinese Glioma Genome Atlas (CGGA) database samples. Clinical outcomes for glioblastoma patients were also taken from the TCGA database.

### Cell proliferation assay

A water-soluble tetrazolium salt (WST-1) assay was conducted to investigate the cell proliferation of U118MG and T98G cell lines following the manufacturer’s instruction (cat No. Ez-500, EZ-Cytox, DoGenBio, Seoul, South Korea). Briefly, 2×10^3^ cells were seeded in 96-well plates. After 24 h of seeding, MCC siRNA or scrambled siRNA transfection was performed. Then, 0 h (immediately after transfection), 24 h, 48 h, and 72 h post-transfection, 10 μl of WST-1 reagent was added. One hour later, the plate was taken to measure absorbance at 450 nm using a microplate reader (EZ Read 800, Biochrom).

### Wound healing assay

U118MG and T98G cells were grown to confluence in six-well plates to determine cell migration using a wound healing assay. A sterile 200-μl yellow pipette tip was used to induce a line “wound” before washing with PBS to discard dead cells and debris. The medium was then replaced with serum-free medium for culturing. Both cells with MCC siRNA or scramble siRNA transfection were observed under a microscope to evaluate the migration of cells. Wound closure was quantified using ImageJ and shown as a percentage of gap closure.

### Statistical analysis

Data are expressed as the mean ± standard error (SE) from at least three experiments performed individually in triplicate. The differences among groups were calculated using Student’s *t*-test and *p*<0.05 was considered significant. Significance was indicated in all figures as follows: *p<0.05 and **p<0.01, compared with the corresponding control values. Quantitative analyses of the results were performed by using Image J software (version 1.52a).

## Results

### MCC is upregulated in glioblastoma and related to poor prognosis

**Figure 1:**
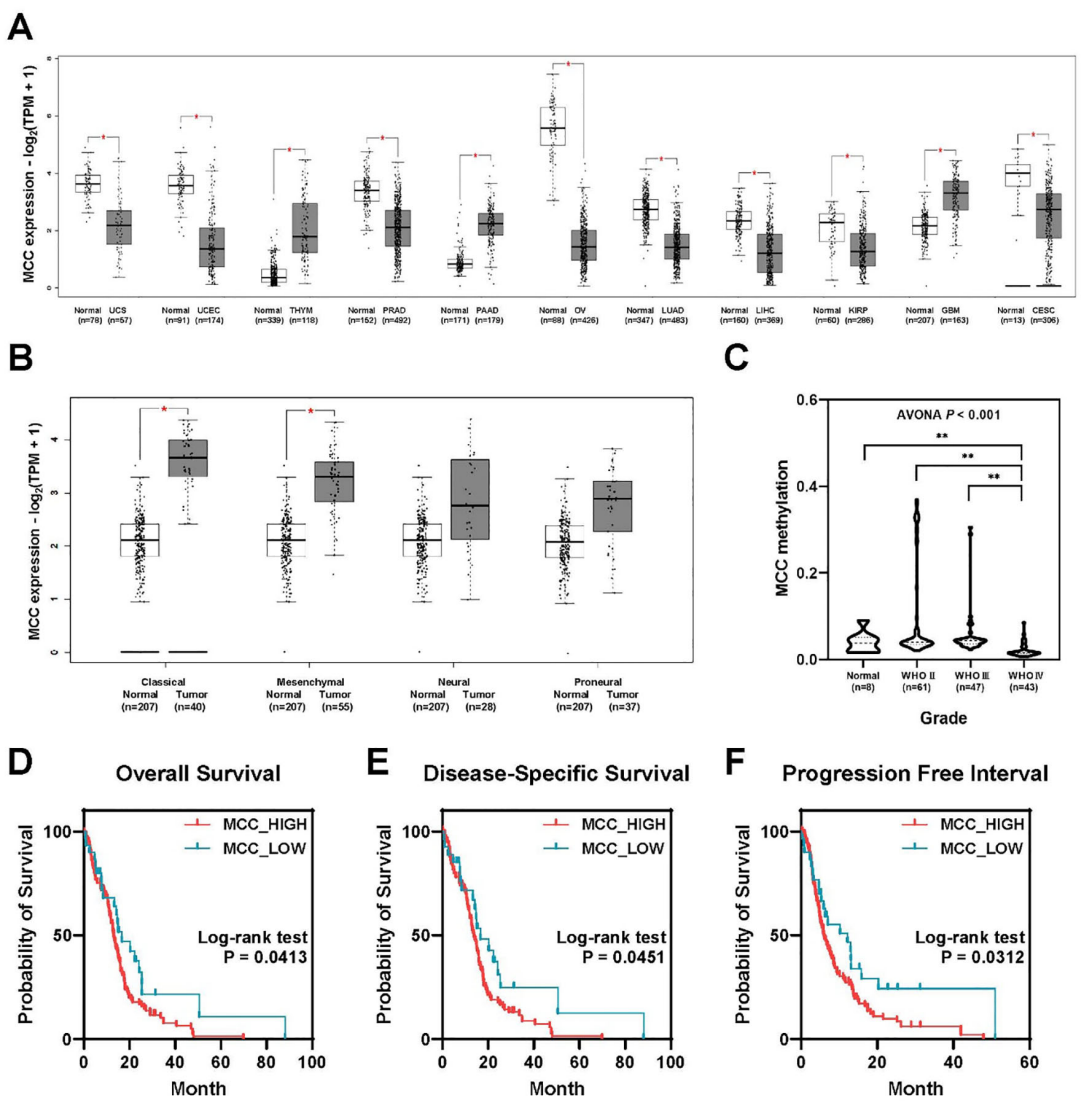
MCC is overexpressed in brain tumors, linked to poor prognosis. **(A)** Differential expression of MCC between tumor and normal tissues in pan-cancer analysis. **(B)** The expression of MCC in GBM subtypes. **(C)** Gene methylation of MCC in different glioma grades. **(D–F)** Kaplan–Meier survival curves comparing the probability of survival between groups with high and low MCC expression across three different metrics: **(D)** overall survival, **(E)** disease-specific survival, and **(F)** progression-free interval. (*p<0.05, **p<0.01).

Analysis of MCC expression in 31 cancer types from TCGA data revealed differential expression patterns between normal and tumor samples in each cancer category. Notably, MCC expression was significantly downregulated in the following eight cancer types: cervical squamous cell carcinoma and endocervical adenocarcinoma (CESC), kidney papillary cell carcinoma (KIRP), liver cancer (LIHC), lung adenocarcinoma (LUAD), ovarian serous cystadenocarcinoma (OV), prostate cancer (PRAD), endometrial cancer (UCEC), and uterine carcinosarcoma (UCS). Conversely, significant upregulation of MCC was observed in the following three cancer types: glioblastoma multiforme (GBM), pancreatic adenocarcinoma (PAAD), and thymoma (THYM). These findings underscore the different roles of MCC in cancer biology; MCC may act as a tumor suppressor or as an oncogene depending on the cancer type (**Figure 1A**). The analysis of gene expression subtypes showed that the MCC expression of classical and mesenchymal types was higher than that of the normal controls (**Figure 1B**). It is known that the frequency of IDH1 mutations is lower in classical and mesenchymal subtypes than in proneural and neural subtypes, which is consistent with the recent WHO classification, in which glioblastomas are classified as IDH wild-type (15). Additionally, analysis of CGGA data revealed that MCC methylation significantly decreases with increasing WHO glioma grade (**Figure 1C**). This reduction in MCC methylation may explain the increased expression of MCC in grade 4 glioblastoma.

Analysis of TCGA-GBM data revealed a significant association between MCC expression levels and clinical outcomes in glioblastoma patients. Surprisingly, patients with high MCC expression exhibited a poorer prognosis than those with low MCC expression (**Figures 1D–F**). The high MCC expression group (N = 129) exhibited lower overall survival rates than the low MCC expression group (N = 30), as shown in **Figure 1D** (log-rank test, *p*=0.0413). Similar to overall survival, the high MCC expression group (N = 119) showed reduced disease-specific survival probabilities compared with the low MCC expression group (N = 27), as shown in **Figure 1E** (log-rank test, *p*=0.0451). The progression-free interval indicates the time until disease progression or recurrence after treatment. The high MCC expression group (N = 129) experienced quicker progression or recurrence than the low MCC expression group (N = 30), as evidenced by the steeper decline in survival probability (**Figure 1F**, log-rank test, *p*=0.0312). These findings suggest that elevated MCC expression may play a role in glioblastoma progression and could serve as an indicator of adverse clinical outcomes.

### MCC is highly expressed in glioblastoma cell lines

**Figure 2:**
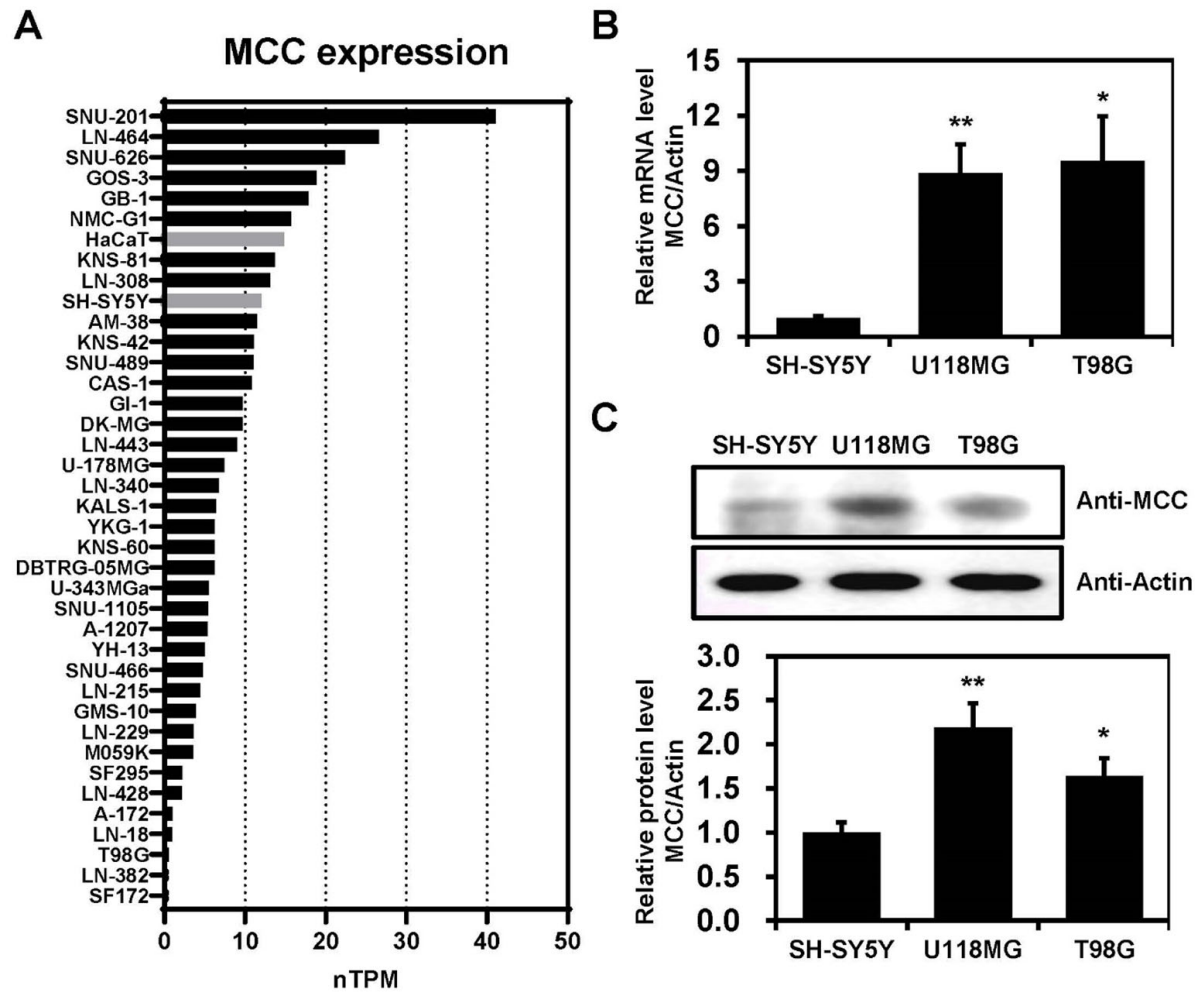
MCC expression in glioblastoma cell lines. **(A)** Bar graph illustrating the MCC expression levels across glioblastoma, SH-SY5Y, and HaCaT cell lines. The data were downloaded from the Human Protein Atlas database and measured in normalized transcripts per million (nTPM). **(B)** Total RNA was extracted from SH-SY5Y and glioblastoma cell lines and *MCC* mRNA levels were measured by qPCR. **(C)** Protein levels of MCC in glioblastoma cell lines (U118MG and T98G) compared with the control cell line (SH-SY5Y). Quantification of the Western blot bands illustrates the protein expression levels of MCC. β-actin was used as the reference gene. Data are mean ± SE (*p<0.05, **p<0.01).

MCC expression levels were investigated across various cell lines, including glioblastoma and non-brain cancer cell lines, such as HaCaT and SH-SY5Y (**Figure 2A**). The data were downloaded from the Human Protein Atlas and measured in normalized transcripts per million (nTPM). The data indicate considerable variability in MCC expression across the cell lines, which may suggest differential roles or regulatory mechanisms of the *MCC* gene in these cell lines. Furthermore, qPCR analyses confirmed that the levels of mRNA encoding MCC were significantly elevated in U118MG and T98G cells compared with SH-SY5Y cells (**Figure 2B**). Immunoblotting analysis using an anti-MCC antibody was performed to investigate the putative roles of MCC in glioblastoma. The level of MCC protein expression was significantly higher in U118MG and T98G cells than in SH-SY5Y cells (**Figure 2C**). Such differential expression highlights the distinct molecular pathology of glioblastoma compared with non-cancerous cells.

### Nuclear localization of MCC in glioblastoma cell lines

**Figure 3:**
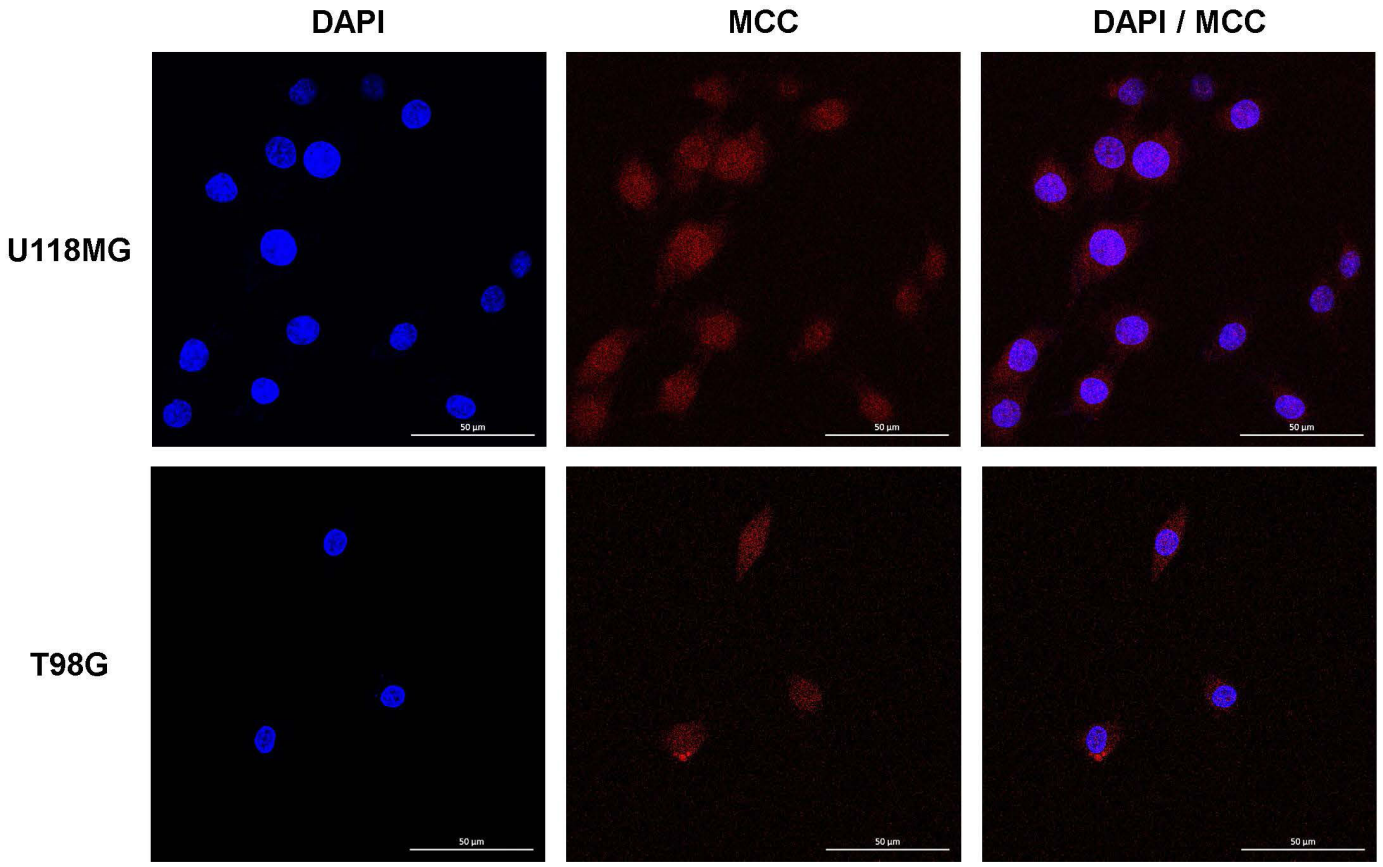
Intracellular localization of MCC in U118MG and T98G cells. Subcellular localization of MCC in cultured cells determined by IF staining. U118MG and T98G cells were stained with anti-MCC antibody. Cell nuclei were stained with DAPI. Cells were imaged by Leica confocal microscopy.

To determine the intracellular location of MCC, U118MG and T98G cells were subjected to immunofluorescence imaging. This revealed MCC predominantly in dot-like structures within the nucleoplasm, with some MCC signal in the cytosol (**Figure 3**). This distinctive pattern suggests a complex role for MCC in cellular functions, potentially implicating MCC involvement in both nuclear and cytosolic processes relevant to glioblastoma pathology.

### Increased MCC expression in tumor tissues from glioblastoma patients

**Figure 4:**
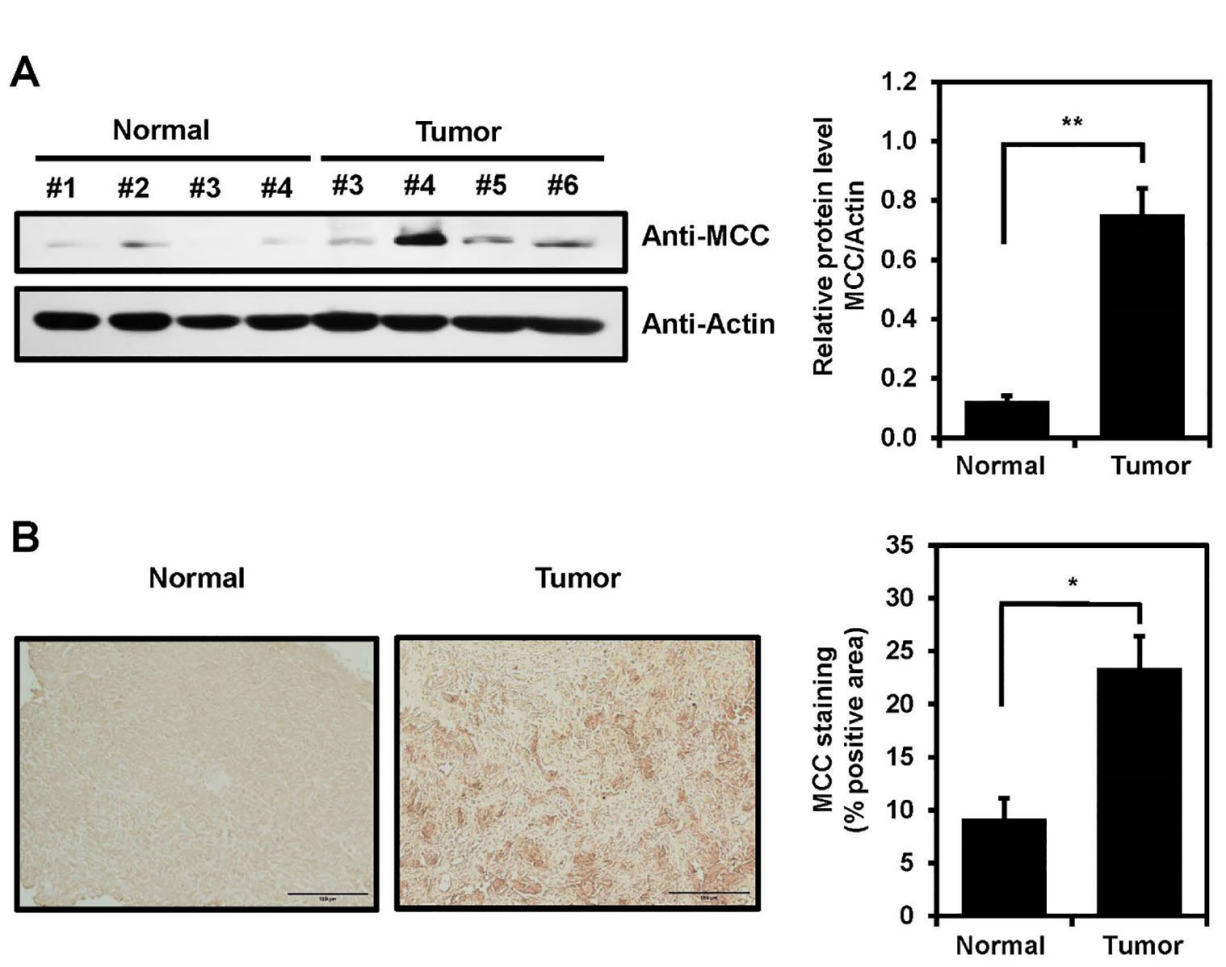
MCC expression in human brain tumor. **(A)** MCC protein levels in glioblastoma tumors compared with normal tissues (left panel). Quantification of the Western blot bands illustrates the protein expression levels of MCC. β-actin was used as the reference gene (right panel). **(B)** Immunohistology staining in glioblastoma tumor and normal tissues with anti-MCC antibody. Quantification of IHC was measured using Image J with n = 3 for normal tissues and n = 5 for glioblastoma tumors. Data are mean ± SE (*p<0.05, **p<0.01).

To explore the possibility that MCC is expressed in clinical brain cancer samples, total tissue lysates of normal and glioblastoma tissues derived from patients during surgery were analyzed by immunoblotting using an anti-MCC antibody. MCC expression in glioblastoma tissues was increased compared with normal tissues (**Figure 4A**). Furthermore, human brain tissues were subjected to immunohistochemical (IHC) analysis to investigate MCC expression levels. Using an antibody against MCC, IHC staining of tumor tissues revealed a significant increase in MCC levels compared with those of normal brain tissues (**Figure 4B**).

### Decreased cell proliferation and migration after MCC knockdown of U118MG and T98G cells

**Figure 5:**
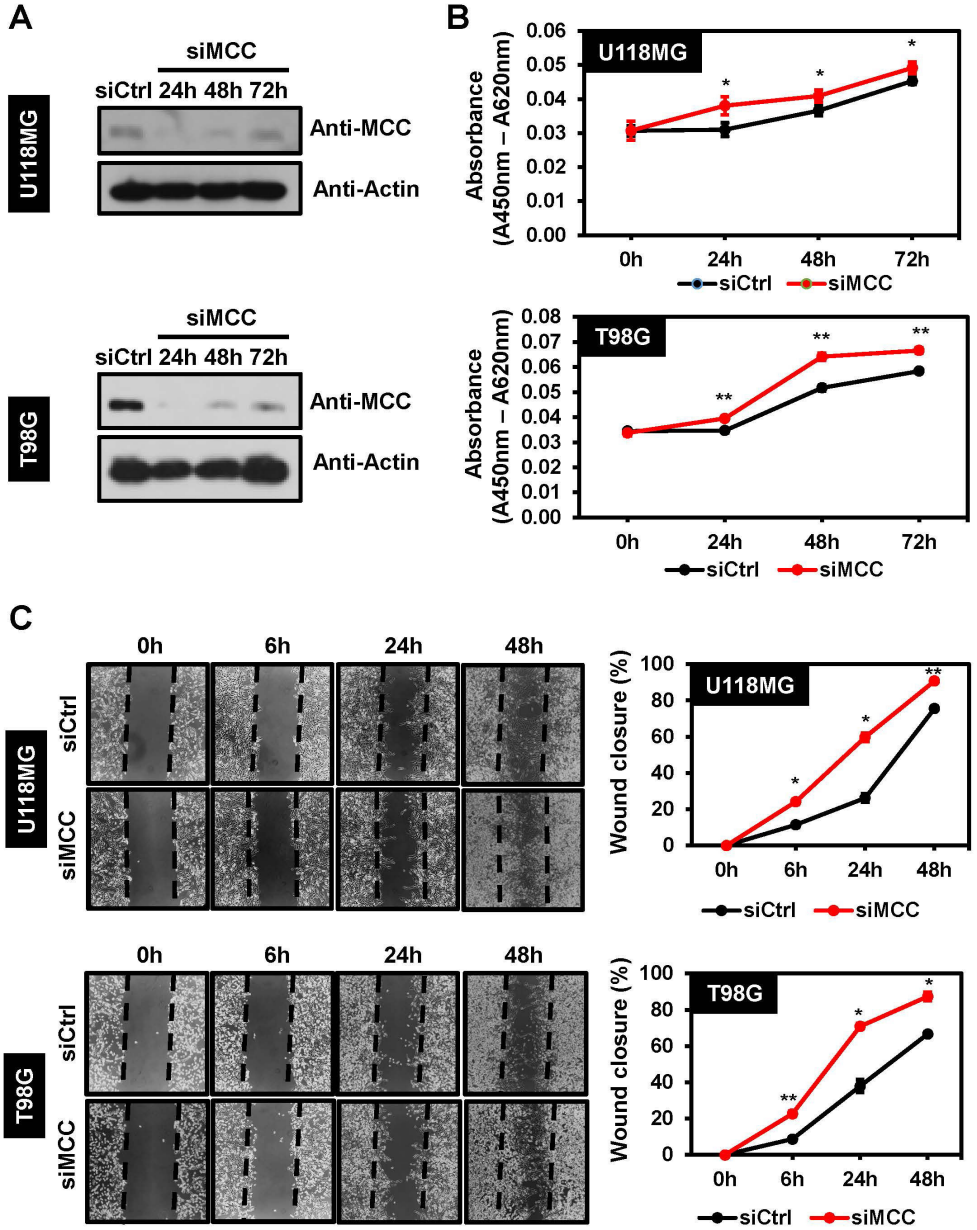
Effects of MCC on cell proliferation and cell migration in glioblastoma cell. **(A)** MCC protein levels 72 h post-transfection with scrambled siRNA (siCtrl) and 24 h, 48 h, and 72 h post-transfection with MCC siRNA in U118MG and T98G cell lines. β-actin was used as the reference gene. **(B)** Line graphs showing cell proliferation evaluated by a WST-1 assay 0 h, 24 h, 48 h, and 72 h post-transfection with MCC siRNA or scrambled siRNA (siCtrl) in U118MG and T98G cell lines. **(C)** Representative images of wound healing assays (left panel) and line graphs showing quantification of relative wound closure (right panel). The wound healing assay was performed and measured after MCC siRNA or scrambled siRNA (siCtrl) transfected cells reached 90% confluent. Data are mean ± SE (*p<0.05, **p<0.01).

To gain deeper insight into the role played by MCC in glioblastoma, the U118MG and T98G cell lines were transfected with MCC-specific siRNA or scrambled siRNA. The scrambled siRNA transfection results for MCC expression were confirmed in U118MG and T98MG cells. There was no difference in MCC expressions during the experiment time at 0 h (immediately after transfection), 24 h, 48 h, and 72 h (**Supplementary Figure 1**). However, MCC protein expression in cells transfected with MCC siRNA was decreased compared with 72-h post-transfected scrambled siRNA as control (**Figure 5A**). Subsequently, the WST-1 and wound-healing assays revealed that the specific suppression of MCC translation significantly increased the proliferation and migration of siRNA-treated cells compared with control cells (**Figures 5B, C**), suggesting that MCC might act as an anti-tumor agent in glioblastoma.

## Discussion

Brain and nervous system cancers account for 1.6% of all new cases and 2.5% of all new deaths caused by all types of cancer (16). Glioblastoma is the most common primary malignant tumor of the adult brain (17). Glioblastoma accounts for >14.5% of all CNS tumors and approximately 48.6% of all malignant CNS tumors (18). When diagnosing glioblastoma, the current primary methods are neuroimaging and histopathological and molecular analyses of tissue biopsies (19). The many treatments include surgery, radiation, and chemotherapy, but the number of biomarkers aiding accurate and early diagnosis remains limited (20). The various biomarkers, the use of which has become routine when clinically diagnosing glioblastoma patients, include O6-methylguanine DNA methyltransferase (MGMT) (21–24), IDH (25–28), EGFR (29–31), TERT (32–34), tumor suppressor protein (TP53) (9, 35), and phosphatase and tensin homolog (PTEN) (36, 37). Further research on biomarkers such as vascular endothelial growth factor (VEGF) (38–40), phospholipid metabolites (41), cancer stem cells markers (42, 43), and various microRNAs (miRNAs) (9, 44) is continuously being conducted. For example, MGMT promoter methylation and TERT promoter mutation improved the overall survival rate (45). Our study provides evidence that *MCC*, a tumor suppressor gene of CRC, might have a distinctive role in the pathophysiology of glioblastoma. We found that MCC was more highly expressed in tumor tissues than in normal brain tissues from glioblastoma patients. Another interesting finding is that MCC of the U118MG and T98G cell lines was expressed in both the nucleoplasm and cytosol, but mainly in the nucleoplasm (**Figure 3**). This result is similar to what was found in the U251MG cell line, another glioblastoma line, as recorded in the Human Protein Atlas (https://www.proteinatlas.org/ENSG00000171444-MCC/subcellular#human). In Purkinje cells and nerves, MCC is found in the plasma membranes and membrane organelles, not in nuclei of the cerebellar cortex (12). Our findings regarding the aberrant subcellular location of MCC in glioblastoma suggest that MCC function and signaling are abnormal in brain tumors compared with normal tissues. In glioblastoma, MCC is translocated from plasma membranes and organelles to the nucleus and then engages in an unknown form of signaling. Further studies are required to confirm this.

In glioblastoma patients, the MCC protein level, as revealed by Western blotting and IHC staining, was significantly higher than in normal tissues (**Figure 4**). Additionally, the bioinformatics data showed that the MCC expression level in glioblastoma tumors was higher than that in normal areas, which may be related to *MCC* gene methylation (**Figure 1**). Contrary to our findings, results from the TCGA-colon adenocarcinoma (COAD) database indicate that MCC expression is lower in the tumor tissues than in adjacent normal tissues. In a subgroup analysis of CRC, MCC was silenced by promoter hypermethylation and was associated with poor prognostic markers, such as high tumor grade and metastasis (46). These findings highlight the dual role of MCC in cancer biology, in which it can function as either a tumor suppressor or an oncogene, depending on the type of cancer.

To further investigate the roles of MCC in glioblastoma, cell proliferation and cell migration assays were conducted, and the results revealed unexpected effects of MCC in glioblastoma cell lines. The expression levels of MCC in glioblastoma cell lines and human tumors were higher than in normal samples (**Figure 5**), but MCC knockdown cells exhibited elevated proliferation and migration, suggesting that MCC serves as a tumor suppressor in glioblastoma. This suggestion is supported by the known effects of MCC in other types of cancer. In detail, MCC was first identified in patients with the autosomal-dominant human hereditary colon cancer syndrome (FAP), with involvement of the adenomatous polyposis coli (*APC*) gene on chromosome 5q (47). MCC was silenced by promoter hypermethylation in a subset of CRC patients (46). Furthermore, transposon-mediated mutagenesis identified *MCC* as a driver gene of carcinogenesis in a mouse model of CRC (48). The re-expression of MCC in a CRC cancer cell line (HCT15) resulted in G2/M arrest, whereas knockdown of MCC attenuated the induction of G2/M arrest under ultraviolet radiation (49). In patients with hepatocellular carcinoma, MCC regulates the oncogenic β-catenin/Wnt signaling pathway, which is often activated in such patients (50). MCC methylation was linked to adverse prognostic markers, high tumor grade, and metastasis in one patient cohort (46). MCC interactions with E-cadherin and β-catenin in the HCT116 cell line were proven via co-immunoprecipitation studies, and MCC ablation promoted HCT116 invasiveness, indicating that MCC serves as a tumor suppressor in the context of the regulation of E-cadherin-mediated cell–cell adhesion of CRC cells (50). The similar effects of MCC on CRC and glioblastoma cells suggest that MCC might influence cell invasiveness by modulating E-cadherin activity; *in vitro* experiments are required to confirm this.

Based on our findings, MCC appears to play a pivotal role in oncogenesis, particularly in glioblastoma. The observed correlation between MCC expression levels and glioblastoma status indicates the MCC may serve as a new biomarker of glioblastoma. Further work is required to define the detailed molecular mechanisms by which MCC modulates glioblastoma pathogenesis.

## Data Availability

The datasets presented in this study can be found in online repositories. The names of the repository/repositories and accession number(s) can be found in the article/**Supplementary Material**.
