# Supplementary figures and images for: The mutated in colorectal cancer (MCC) gene can serve as a potential biomarker of glioblastoma
Source: Front Oncol. 2024 Oct 8;14:1435605. doi: 10.3389/fonc.2024.1435605 (PMC11493605; doi:10.3389/fonc.2024.1435605)

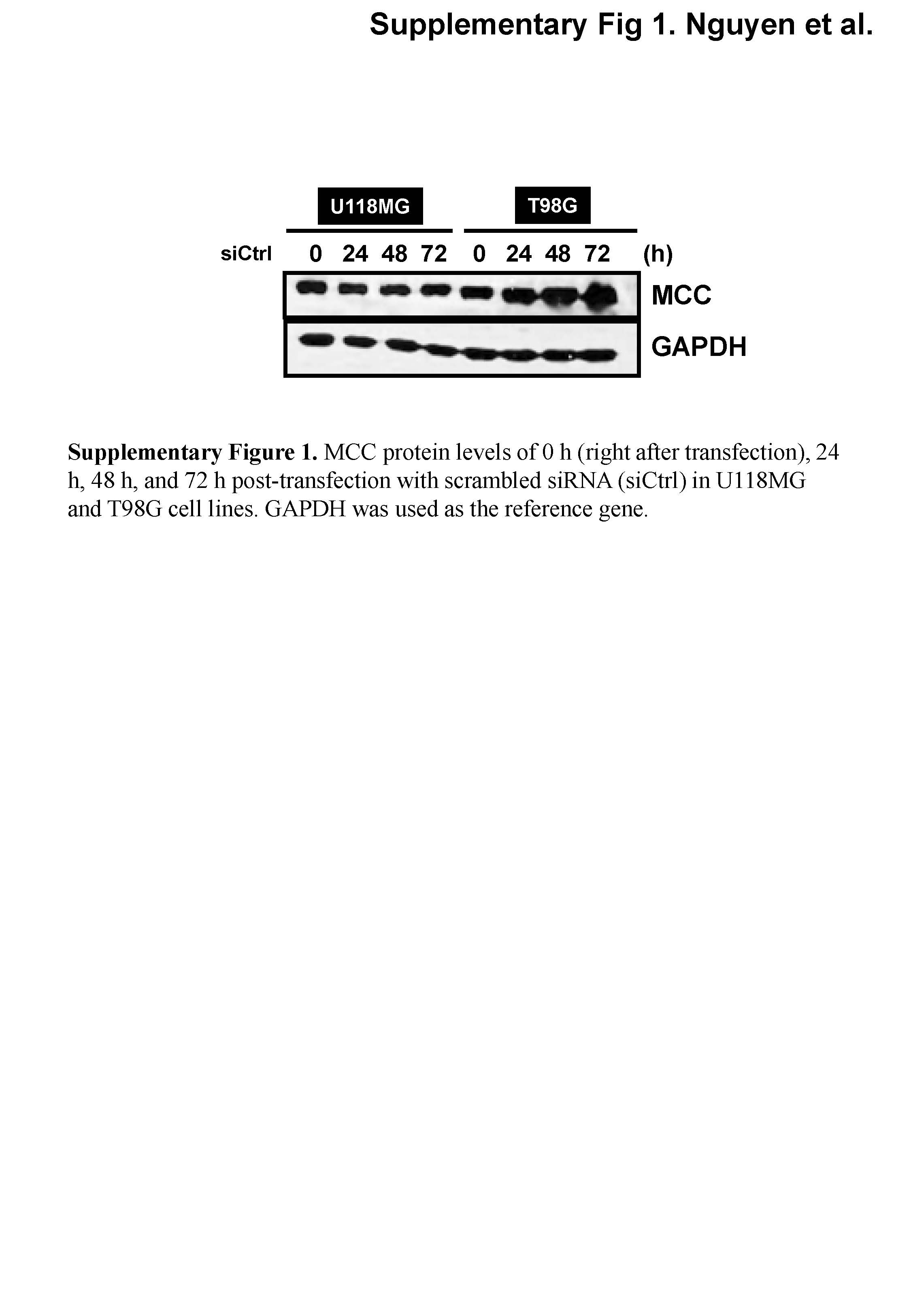

Supplement: Supplementary file 1 [file Image1.jpeg]
